# A Novel Multi-Region, Multi-Phase, Multi-Component-Mixture Modeling Approach to Predicting the Thermodynamic Behaviour of Thermoplastic Composites during the Consolidation Process

**DOI:** 10.3390/polym14214785

**Published:** 2022-11-07

**Authors:** Eva Kobler, Janos Birtha, Christian Marschik, Klaus Straka, Georg Steinbichler, Paul Zwicklhuber, Sven Schlecht

**Affiliations:** 1 Competence Center CHASE GmbH, Altenberger Straße 69, 4040 Linz, Austria; 2 Intitute of Polymer Injection Moulding and Process Automation Linz, Johannes Kepler University, Altenberger Straße 69, 4040 Linz, Austria; 3 ENGEL AUSTRIA GmbH, Steyrer Straße 20, 4300 St. Valentin, Austria; 4 Covestro Deutschland AG, Kaiser-Wilhelm-Allee 60, 51373 Leverkusen, Germany

**Keywords:** thermoplastic composites, processing, consolidation, modelling, CFD

## Abstract

In the processing of thermoplastic composites, great importance is attributed to the consolidation step, as it can significantly reduce the porosity of the semi-finished product and thus influence considerably the quality of the final component. This work presents an approach to modeling the thermodynamic behavior of composite materials during hot-press consolidation. For this purpose a multi-region, multi-phase and multi-component-mixture model was developed using the simulation toolbox OpenFOAM^®^. The sensitivity of the model was tested by varying the thermal parameters and mesh resolution, confirming its robustness. Validity of the model was confirmed by comparing simulation results to experimental data for (i) polycarbonate with 44% carbon fiber by volume and (ii) polypropylene with 45.3% glass fiber by volume. The simulation allows very precise estimation of when a particular temperature, such as the glass transition temperature or melting point, will be reached at the core of a composite. In relation to the total process time, maximum deviation of the simulation from the experimental data amounted to 2.84%. Therefore, the model is well suited for process optimization, it offers a basis for further model implementations and the creation of a digital twin.

## 1. Introduction

Achieving climate targets, such as a reduction in CO_2_ emissions, requires efficient solutions in the transportation sector, first and foremost in the aviation and automotive industries. Lighter components with consistently good mechanical properties are used to reduce fuel and energy consumption and thus lower harmful emissions. Composite materials that consist of a polymer matrix with fiber reinforcement have been shown to fulfill these requirements [1,2,3,4].

Modern development of composites gained traction in the 1930s with the introduction of glass fibers by the Owens-Illinois glass company and with patents awarded to Carlton Ellis and Paul Schlack for polyester and epoxy resins, respectively. It was found that combining polymer resin with glass fiber resulted in materials with excellent mechanical properties at low weight; these materials were further improved during World War II, which led to their increased commercial availability and production in the USA [5].

Research into thermoplastic matrix systems began in the 1980s, but only became significant when thermoplastic composites were used in the construction of the Airbus A340-600 in 2002: Glass-fiber reinforced thermoplastics were employed to form most of the inboard leading edge and turned out not only to be 20% lighter, but also more resistant to impact damage and more repairable, than the aluminum they replaced. Airbus therefore used thermoplastic composites in two thirds of the fixed leading edges for their A380 [6,7,8].

Polymer-based composites can be classified as thermoset or thermoplastic, the main difference being the crosslinking in the matrix phase. While thermoset matrix systems, such as epoxy, phenolic, polyesters and vinyl ester resins, fully crosslink during the curing process, thermoplastic polymers, such as polyolefines, polycarbonate (PC), polyamide (PA), and polyaryletherketons (PAEK), exhibit minimal crosslinking. The temperature-dependent molecular chain mobility of thermoplastics renders mechanical and rheological processes applied to them reversible, which makes thermoplastic composites reprocessable, allows functional integration by overmolding and supports repairability by welding and recyclability [1]. This leads to several advantages thermoplastic composites offer over thermoset composites: (i) high damage tolerance in terms of high fracture toughness, (ii) high impact and fatigue resistance, and (iii) outstanding corrosion and solvent resistance. Since no curing reaction takes place in thermoplastic composites, processing is fast and automatable, storage is relatively cheap, and shelf life is unlimited. However, thermoplastic composites require higher temperatures and pressures during processing than thermoset composites which may result in higher manufacturing costs [9,10,11,12,13].

According to the JEC [14] and IMARC Groups [15], the global market for composites in 2021 was estimated at approximately 12 Mtons and US$ 37 billion, US$ 16.1 billion of which were thermoplastic composites. The IMARC Group projected that the market for thermoplastic composites will reach a value of US$ 23.3 billion by 2027, with a compound annual growth rate of 6.25% over the period from 2022 to 2027. The largest customer for thermoplastics is the automotive industry [16].

**Figure 1:**
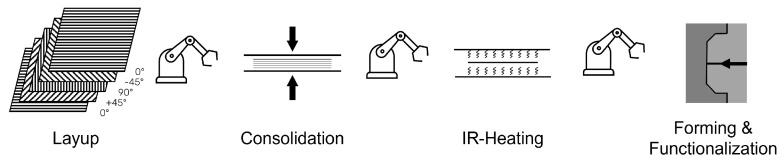
Processing of thermoplastic UD tapes by laying, consolidation, preheating, forming and functionalization.

Processing techniques differ depending on fiber length (short, long or continuous) and on whether the reinforcement is realized by single fibers or textiles (e.g., woven or braided). The processing of thermoplastic unidirectional continuous fiber reinforced (UD) tapes relevant to this work consists of the following steps: Tape laying, consolidation, preheating, forming and functionalization, as shown in Figure 1.

During tape laying, the individual tapes are stacked on top of each other. Since UD tapes are highly loadable only in the fiber direction, the fiber orientation is usually varied during laying. For aerospace applications, a layup of $[0^{\circ }|\pm 45^{\circ }{\left|90^{\circ }\right]}_{S}$ is prominent [17]. Tape laying can be fully automated: either pick-and-place, automated tape laying (ATL), or automated fiber placement (AFP). In pick-and-place tape laying, the individual pre-cut tapes are stacked on top of each other by a robot and welded locally, for instance, by hot-stamping or ultrasonic welding. Since the pick-and-place principle involves welding the tapes together locally, subsequent consolidation is required. The consolidation process can be carried out using hydraulic heating and cooling presses, where the layup is first heated under pressure in a heating press beyond the glass transition or melting point of the matrix material and then cooled in a cooling press, again while applying pressure, to a temperature below the glass transition or melting point of the matrix material.

In order to be formable the consolidated part must subsequently be heated to a temperature above the melting point for semi-crystalline polymers and above the glass transition temperature for amorphous polymers. Infrared or convection ovens are usually used in the preheating process [18], during which deconsolidation may occur depending on the properties of the product, such as the degree of porosity, moisture content, fiber network crosslinkage, degree of crystallization and matrix viscosity [19,20].

The forming process is often carried out by hydraulic presses, in which a semi-finished product is held between the mold halves and formed when they are brought together. This process can also be carried out in an injection molding machine, which allows simultaneous overmolding and thus functionalization.

As mentioned above, consolidation of sufficient quality can be achieved without a specific consolidation step if optimal process parameters are used for in-situ consolidation during the ATL or AFP process. However, a consolidation step by using a pressing operation before pre-heating and forming increases quality in terms of porosity and interlaminar shear strength (ILSS) [21].

As described in [22], ATL and AFP are mostly used during the production of thermplastic composite parts. Hence, lastest literature about the consolidation process and its modeling is related to ATL and AFP, whereas hardly any literature can be found on the consolidation process using a hot press. This work focuses exclusively on the consolidation by hot press. To predict the thermodynamic behavior of the tape stack during consolidation, we formulated and solved numerically a new mathematical model, using the open-source CFD (Computational Fluid Dynamics) Software OpenFOAM^®^. To this end, we generated a new solver that represents a multi-region, multi-phase and multi-component-mixture flow of a compressible fluid under non-isothermal, transient conditions.

## 2. Modeling

### 2.1. Theoretical Background

**Figure 2:**
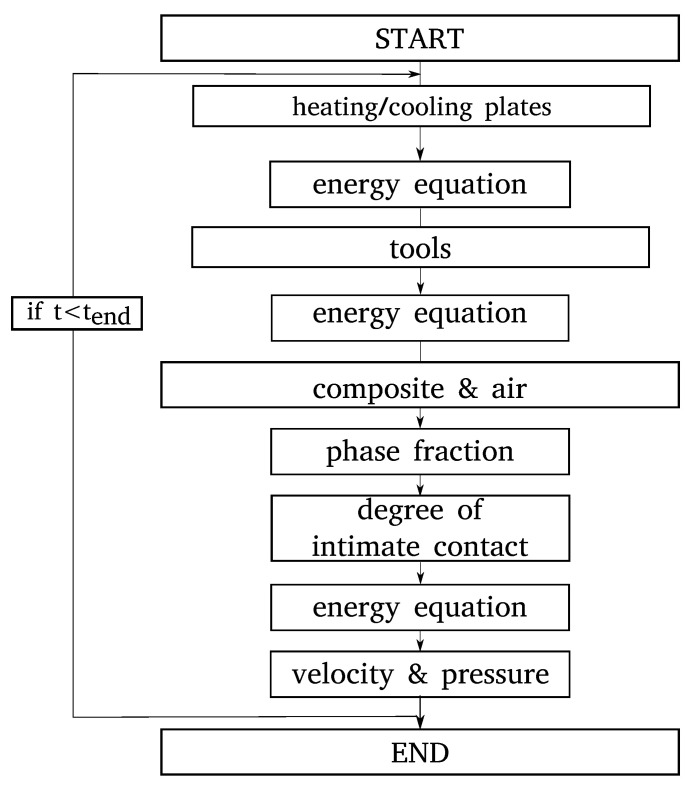
Flowchart for modeling of the consolidation process.

Figure 2 shows the steps of the solution algorithm. The individual equations are described in detail in Section 2.1.1.

#### 2.1.1. Basic Equations

**Figure 3:**
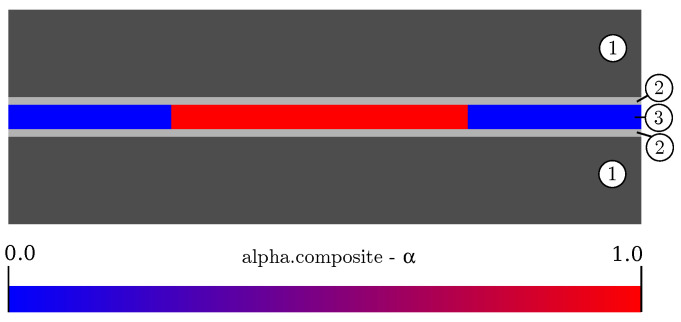
Multi-region and multi-phase domains. The heating/cooling plates (1) and the tools (2). The fluid domain (3) is treated as a multi-phase region that includes a composite part (red) and air (blue).

To predict the consolidation process of composite materials, a mathematical model was developed and solved numerically in OpenFOAM^®^. Figure 3 shows a schematic of the model setup, which comprises a solid and a fluid region. The former includes the heating/cooling plates, which heat/cool and exert pressure to the composite, and the tools needed for transportation between the heating and cooling press. The fluid region consists of two phases: (i) the composite material and (ii) the air that surrounds it laterally. The composite material is represented by a homogeneous multi-component-mixture model that comprises polymer matrix and fiber fraction, and ignores the individual tape layers. Thus, a model of a compressible fluid flow under transient, non-isothermal conditions is obtained.

Division of the computational grid into solid and fluid domains means that different assumptions are made for the respective regions and different equations are solved. For the solid domains, including the heating/cooling plates and the tools, the energy conservation equation is solved based on pure heat conduction.

(1) $$\frac{\partial \rho h}{\partial t}=\nabla \cdot \left(\alpha_{th}\nabla \cdot h\right).$$

Equation (1) describes the change in specific enthalpy *h* over time as a function of thermal diffusivity $\alpha_{th}$

(2) $$\alpha_{th}=\frac{\lambda }{\rho *c_{p}},$$

where λ is the heat conductivity, ρ the density, and $c_{p}$ the specific heat capacity at constant pressure. The heating and cooling plates and the tools are made of steel, for which the relevant values were obtained from the literature: λ: 54 W/(m*K), ρ: 7854 kg/m^3^, $c_{p}$: 461 J/K.

The transport of the phases in the fluid area (composite and air) is generally described by the conservation equations of mass, momentum, and energy in combination with the Volume of Fluid (VOF) model (see Equations (3), (4), (5) and (8))

(3) $$\frac{\partial \alpha }{\partial t}+\nabla \cdot \left(\vec{u}\alpha \right)+\nabla \cdot \left({\vec{u}}_{r}\alpha \left(1-\alpha \right)\right)=0,$$

where α is a dimensionless parameter that indicates, whether a cell contains composite (α=1) or air (α=0) (see Figure 3). The last term corrects for the smearing of the two immiscible phases (i.e., 0<α<1), with ${\vec{u}}_{r}$ directed against the flow [23].

Mass, momentum and energy conservation (Equations (4), (5) and (8)) are solved for both phases composite and air in combination with Equation (3), considering the respective material parameters:

(4) $$\frac{\partial \rho }{\partial t}+\nabla \cdot \left(\rho \vec{u}\right)=0,$$

(5) $$\frac{\partial \rho \vec{u}}{\partial t}+\nabla \cdot \left(\rho \vec{u}\vec{u}\right)={\vec{F}}_{B}+{\vec{F}}_{S},$$

with ${\vec{F}}_{S}$ representing surface forces:

(6) $${\vec{F}}_{S}=-\nabla p+\nabla \cdot {\underline{S}}_{vis},$$

where ${\underline{S}}_{vis}$ is the viscous stress tensor, and *p* the pressure. Outer body forces ${\vec{F}}_{B}$, such as gravity, are ignored in this work. Further, to express the viscous stress tensor (Equation (7)), Newtonian behavior is assumed, from which follows that the dynamic viscosity $\eta_{M}$ of the mixture of fiber and matrix is independent of the shear rate. However, the viscosity is assumed to be a polynomial function of the temperature *T* fitted to experimentally obtained data and Equation (9), and is calculated from:

(7) $${\underline{S}}_{vis}=\eta_{M}\left[\nabla \vec{u}+{\left(\nabla \vec{u}\right)}^{T}-\frac{2}{3}\nabla \cdot \vec{u}\underline{I}\right],$$

where η is the dynamic viscosity and $\underline{I}$ the identity tensor. To include non-isothermal effects, the conservation of the inner energy *e* is considered by:

(8) $$\frac{\partial \rho e}{\partial t}+\nabla \cdot \rho \vec{u}e---\left[\nabla \cdot \vec{u}p\right]+\frac{\partial \rho K}{\partial t}+\nabla \cdot \rho \vec{u}K=\nabla \cdot \alpha_{th,eff}\nabla e+\rho S.$$

The first three terms describe the change in inner energy *e* with time, convection of the inner energy *e* and compression heating. The remaining terms represent the change in mechanical energy *K* with time and convection of the mechanical energy *K*, respectively. The terms on the right-hand side describe the heat conduction, with $\alpha_{th,eff}$ being the effective thermal diffusivity. The optional source term *S* allows crystallization or melting energy to be included. However, since the focus was on polycarbonate, an amorphous matrix material, this effect was omitted in this work.

The material properties of the mixture of matrix and fibers, that is, density $\rho_{M}$, thermal conductivity $\lambda_{M}$, specific heat capacity $c_{p,M}$, and dynamic viscosity $\eta_{M}$, are calculated using the rule of mixture (Equation (9)) [24]:

(9) $$\left(\begin{array}{c} \rho_{M}\\ \lambda_{M}\\ c_{p,M}\\ \eta_{M} \end{array}\right)=fvf\left(\begin{array}{c} \rho_{m}\\ \lambda_{m}\\ c_{p,m}\\ \eta_{m} \end{array}\right)+\left(1-fvf\right)\left(\begin{array}{c} \rho_{f}\\ \lambda_{f}\\ c_{p,f}\\ \eta_{f} \end{array}\right)$$

Here, indices *m* and *f* refer to the matrix and the fiber, respectively. Further, fvf is the fiber volume fraction with *V* as the volume:

(10) $$fvf=\frac{V_{f}}{V_{m}+V_{f}}.$$

The density of the matrix $\rho_{m}$ was measured by a high capillary rheometer (HKR Rheograph 25, GÖTTFERT Werkstoff-Prüfmaschinen GmbH, Buchen, Germany). The thermal conductivities of the matrix $\lambda_{m}$ and the fibers $\lambda_{f}$ were taken from literature. The specific heat capacity of the matrix $c_{p,m}$ was determined by differential scanning calorimetry with a Mettler Toledo DSC 1 (Mettler Toledo Group, Columbus, OH, USA), and the dynamic viscosities of the matrix $\eta_{m}$ and the mixture $\eta_{M}$ were evaluated with an ANTON-Paar MCR 302 plate-plate rheometer (Anton Paar, Graz, Austria). The sources of the data are listed in Table 1.

In order to study the effect of varying thermal conductivity and specific heat capacity on the simulation results, a sensitivity study was conducted (see Section 2.2.1). The multi-mixture model describes the composite as a homogeneous material and ignores any local irregularities, such as fiber accumulations. Furthermore, anisotropic thermal and flow behaviors, which heavily depend on the fiber orientation, were ignored.

#### 2.1.2. Boundary Conditions

The boundary conditions have been chosen to the best of our knowlegde to match the reality as exactly as possible. To model the heat transfer between heating/cooling plates, tools and composite, a boundary condition for the heat conduction is used. To this end, a value fraction vf, is calculated at the interface of two regions (solid/solid or solid/fluid), and used to determine the wall temperature $T_{w}$:

(11) $$vf=D_{IC}\frac{\frac{\lambda_{F}}{d_{F}}}{\frac{\lambda_{F}}{d_{F}}+\frac{\lambda_{S}}{d_{S}}}=\left\{\begin{array}{c} 0\to T_{w}=T_{F}.\\ 1\to T_{w}=T_{S}. \end{array}\right.\;$$

The indices *F* and *S* refer to the fluid and solid regions, respectively, and *d* describes the height of a finite volume element at the corresponding boundary wall.

An impeded heat transfer due to surface roughness is considered by the degree of intimate contact $D_{IC}$ in Equation (11), which is based on the work presented in [25,26], and described in [27,28,29,30,31,32] and calculated by:

(12) $$D_{IC}=\frac{1}{1+\frac{w_{0}}{b_{0}}}{\left[1+5\left(1+\frac{w_{0}}{b_{0}}\right){\left(\frac{a_{0}}{b_{0}}\right)}^{2}\int_{0}^{tc}\frac{P_{app}\left(t\right)}{\eta_{0}\left(T\left(t\right)\right)}dt\right]}^{\frac{1}{5}}.$$

**Figure 4:**
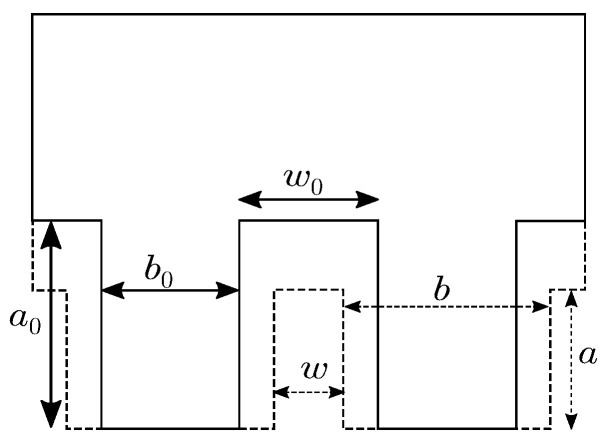
Development of intimate contact and the corresponding geometric parameters, based on the theory of [31].

Intimate contact is based on a simplified view of the surface roughness, which is assumed to be approximately rectangular and described by the initial geometrical values $w_{0}$, $b_{0}$ and $a_{0}$, the applied pressure $P_{app}$ and the temperature-dependent dynamic zero-viscosity $\eta_{0}\left(T\right)$. Figure 4 shows a schematic of the contact region.

Assuming that there is no ideal contact between the heating/cooling plates and the tools, which limits heat conduction, a thin layer of air is considered in the boundary condition, which yields:

(13) $$vf=D_{IC}\frac{\frac{\lambda_{F}}{d_{F}}}{\frac{\lambda_{F}}{d_{F}}+\frac{\lambda_{A}}{d_{A}}}=\left\{\begin{array}{c} 0\to T_{w}=T_{F},\\ 1\to T_{w}=T_{A} \end{array}\right.\;$$

where the index *A* refers to the air layer.

To represent the wall adhesion of the composite material to the tools, a noslip boundary condition is chosen at the interface to exclude velocity along the boundary face. A constant value is specified for the pressure at the boundary to the lower tool; for all other interfaces the pressure is calculated from the velocity.

### 2.2. Simulation Studies

#### 2.2.1. Sensitivity Study

The specific heat capacity of the matrix material $c_{p,m}$ was measured by differential scanning calorimetry (Equipment: Mettler Toledo DSC 1), while the specific heat capacity of the fibers $c_{p,f}$, thermal conductivity of the matrix $\lambda_{m}$ and thermal conductivity of the fibers $\lambda_{f}$ were taken from the literature. To determine the sensitivity of the simulation to these thermodynamic material parameters, a parametric study was carried out. For this purpose, a 10-layer UD tape layup consisting of polycarbonate with 44% carbon fiber by volume was heated from 60 °C to 250 °C within 60 s. For the specific heat capacities $c_{p,m}$, $c_{p,f}$ and the thermal conductivities $\lambda_{m}$, $\lambda_{f}$ we additionally used values that were 20% higher and lower than those taken from the literature. All values used are listed in Table 2.

#### 2.2.2. Mesh Study

**Figure 5:**
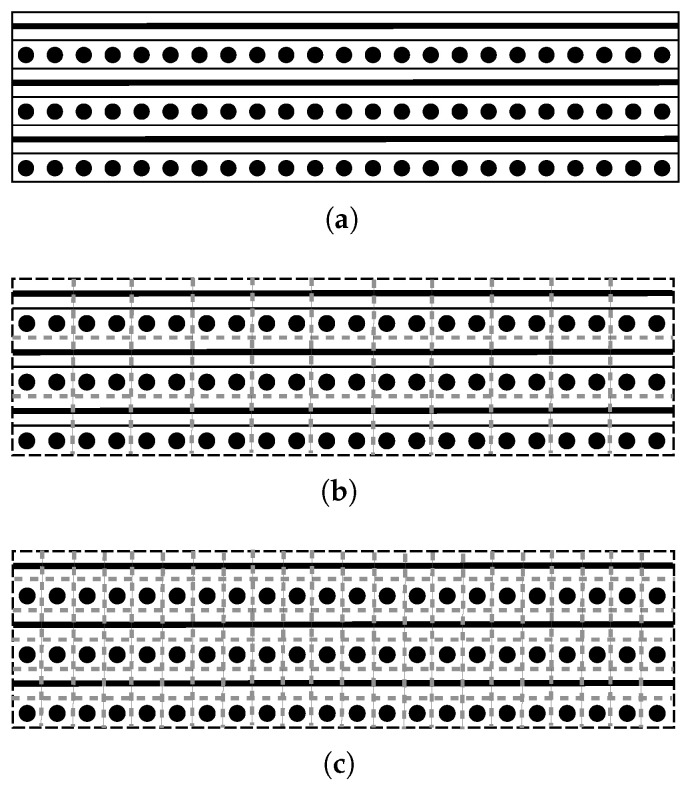
Example for a 6 layer layup (**a**), where the dashed gray lines indicate the cells of a coarse mesh (**b**) and of a fine mesh (**c**).

A mesh study was additionally carried out to assess the influence of cell size distribution on heat transfer. Again, a 10-layer UD tape layup consisting of polycarbonate with 44% carbon fiber by volume was heated from 60 °C to 250 °C within 60 s. Both a coarse and a fine mesh were investigated. The coarse mesh had a cell size of 0.35 mm in the thickness direction; which means that one cell consisted of two tape layers. For the fine mesh the cell size was halved, and thus each cell in the thickness direction represented one layer of tape, as illustrated in Figure 5.

The coarse and fine meshes consisted of a total of 1.5 million and 2.2 million cells, respectively.

#### 2.2.3. Validation Study

**Figure 6:**
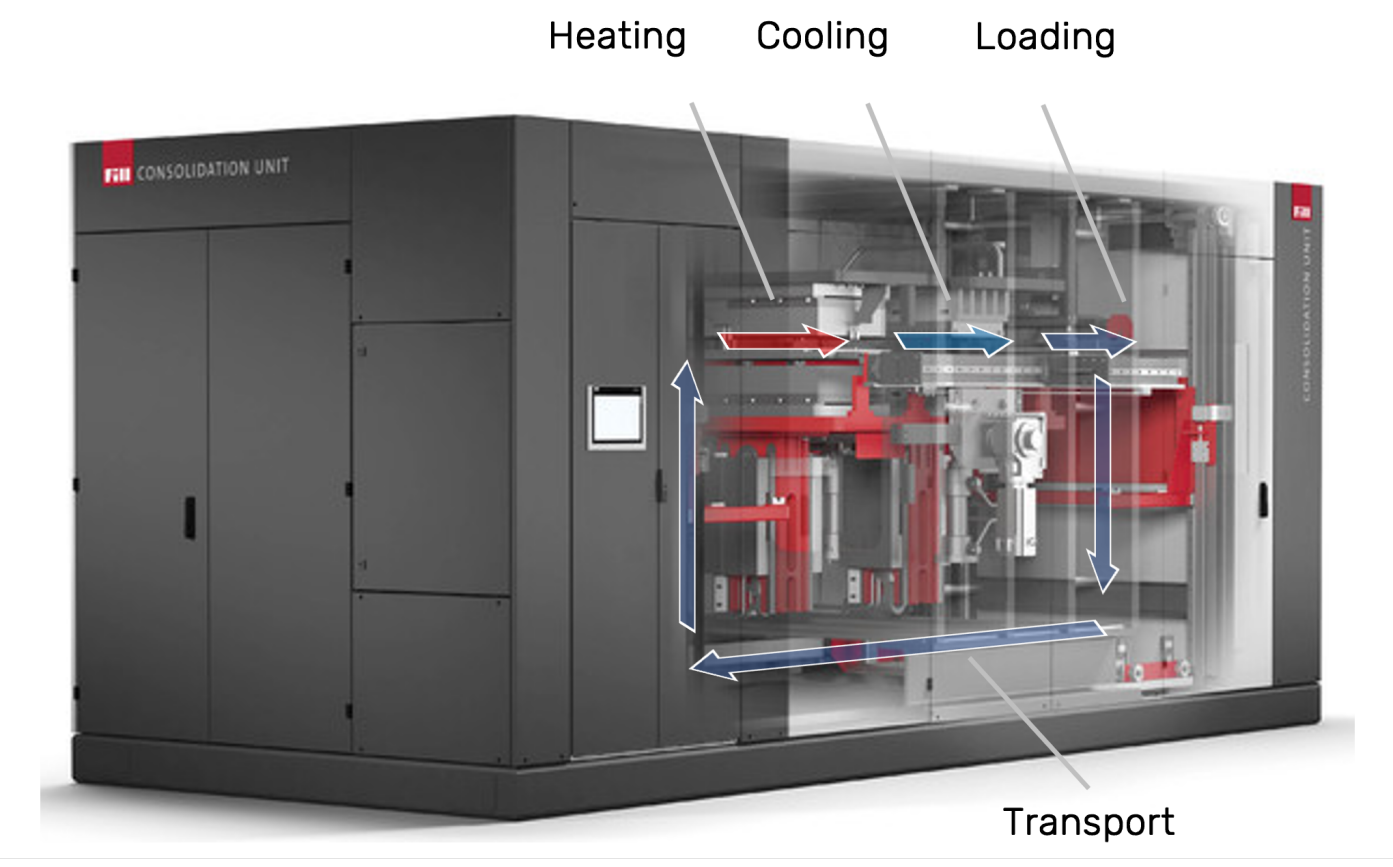
Consolidation unit used for experimental validation (Adapted with permission from [33]. 2022, FILL GESELLSCHAFT M.B.H.).

The consolidation process modeled in this work is carried out by a consolidation unit which includes a heating and a cooling press. Within the consolidation unit, the tape layup is transferred from one press to the other between two steel plates, which are moved within the machine fully automatically (see Figure 6).

**Figure 7:**
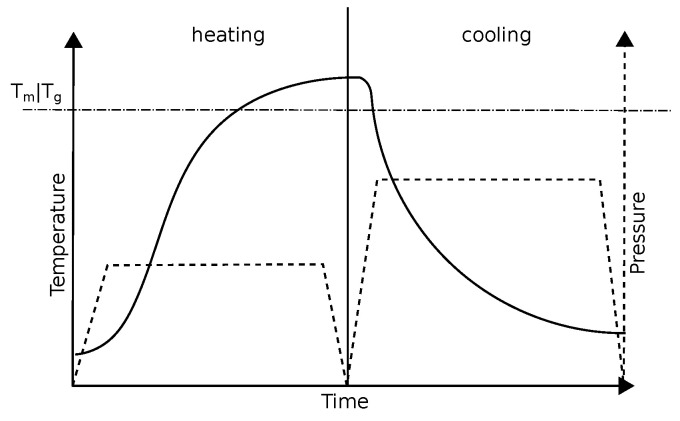
Example temperature and pressure profiles of a random composite material during the heating and cooling phases of a consolidation process.

During the heating process, less pressure is usually applied to the tape layup compared to the cooling process, to avoid extensive squeeze flow. Typical temperature and pressure profiles for this process can be seen in Figure 7.

To analyze the validity of the simulation approach, an experimental parameter study was carried out with various temperature and pressure profiles using various materials, and the results were compared to those of the simulation. The process parameters considered are listed in Table 3.

For case 1, two previously consolidated sheets were consolidated with each other, while in cases 2–4, 18 individual tapes were consolidated into one sheet. The material used for cases 1–5 was a polycarbonate with 44% carbon fiber by volume (PC-CF). In case 5, a 10-layer tape layup of polypropylene with 45.3% glass fiber by volume (PP-GF) was used and only the heating process was investigated.

In order to record the temperature within the composite part, a thermocouple (TC Type K) was placed between the sheets in case 1. In cases 2 to 5, three individual thermocouples were placed between the individual tape layers. In cases 2-4, they were placed between layers 1 and 2, 9 and 10, and 17 and 18. In case 5, they were placed between layers 1 and 2, 5 and 6, and 9 and 10. This made it possible to obtain a complete picture of the temperature behavior at multiple points in the composite and thus allowed comprehensive comparison between experiment and simulation.

## 3. Results

### 3.1. Model Sensitivity to Changes in Thermal Material Properties

**Figure 8:**
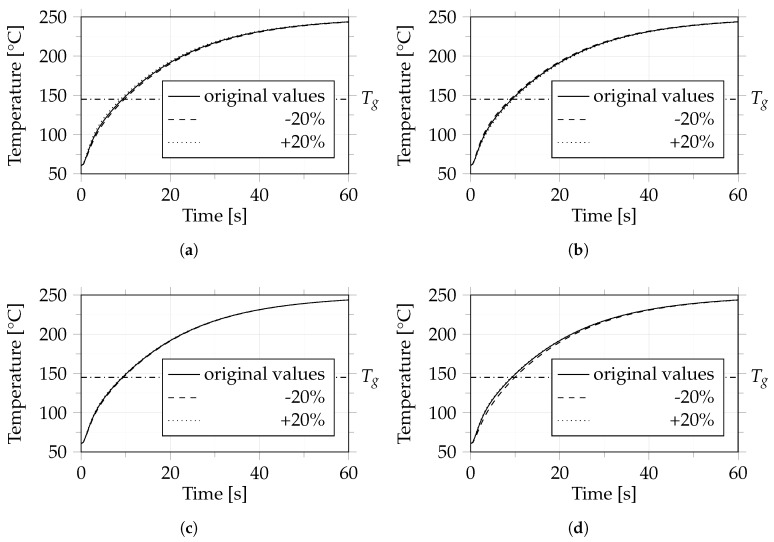
Temperature behavior of the considered composite during heating for various values of specific heat capacity of matrix $c_{p,m}$ (**a**), specific heat capacity of fiber $c_{p,f}$ (**b**), thermal conductivity of matrix $\lambda_{m}$ (**c**) and thermal conductivity of fiber $\lambda_{f}$ (**d**). The dash-dotted line indicates the glass transition temperature ($T_{g}$) of 145 °C.

In the sensitivity study, each thermal parameter (i.e., specific heat capacities $c_{p,m}$, $c_{p,f}$ and thermal conductivities $\lambda_{m}$, $\lambda_{f}$ (see Table 2)) was varied separately by ±20% while keeping all other thermal parameters at their original values from the literature. The results of this study are shown in Figure 8. Due to the large temperature difference between the heating plates and the composite material, the temperature increased sharply at the beginning and reached a plateau after about 60 s. For all thermodynamic parameters, the numerically calculated temperature curves hardly changed between the different settings. This suggests that, within a specific range, the thermal parameters of the individual components—that is, specific heat capacity $c_{p,m}$, $c_{p,f}$ and thermal conductivity $\lambda_{m}$, $\lambda_{f}$—have little influence on the heat transfer.

### 3.2. Mesh Study

**Figure 9:**
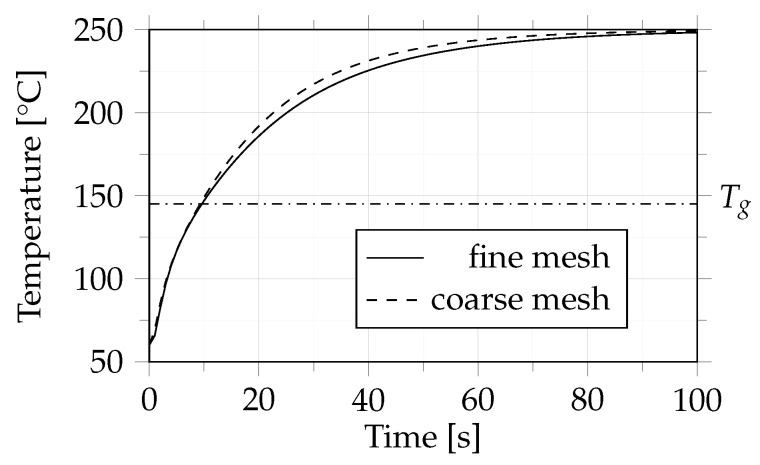
Temperature of considered composite for two meshes; coarse: 1.5 million cells and fine: 2.2 million cells. The dash-dotted line indicates the glass transition temperature ($T_{g}$) of 145 °C.

For the mesh study, a 10-layer composite material that consisted of polycarbonate with 44% carbon fiber by volume was heated from 60 °C to 250 °C. The meshes were defined as described in Section 2.2.2. The coarse mesh consisted of 1.5 million cells and the fine mesh of 2.2 million cells. For comparison, the temperature in the center of the composite part over time was investigated, as illustrated in Figure 9.

The results of this study indicate that the mesh resolution has minimal influence on the temperature behavior. The largest difference between the temperature curves amounted to 6.6 °C after 30 s of heating, which corresponds to a difference in temperature of about 3%. The influence of mesh resolution on other phenomena that occur during consolidation, which were ignored in this work, (e.g., degree of bonding and squeeze flow) remains to be investigated. To save computational time, the subsequent parameter study was conducted using the coarse mesh.

### 3.3. Parameter Study

As described in Section 2.2.3 a parameter study was carried out to test our model’s prediction accuracy against data from experiments using the consolidation unit shown in Figure 6. Each experiment was performed three times to record and minimize inconsistencies between runs, and the mean values were used to compare experiment with simulation.

#### 3.3.1. Case 1

**Figure 10:**
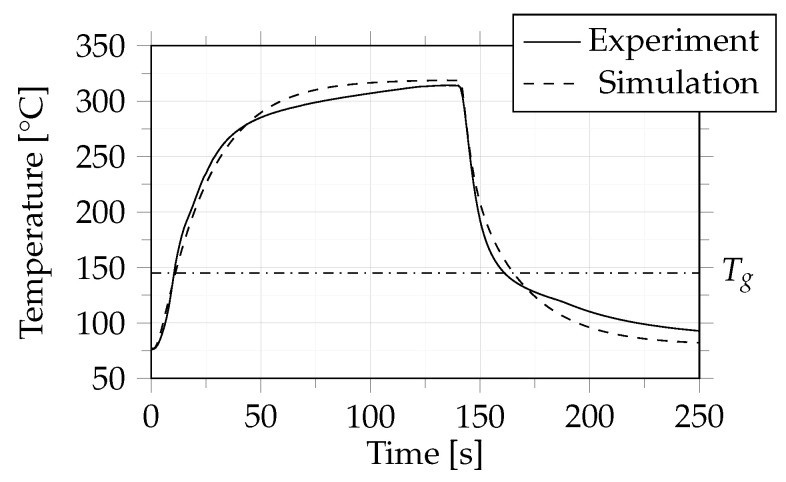
Case 1: Comparison between experiment and simulation in terms of temperature behaviour of the composite. The dash-dotted line indicates the glass transition temperature ($T_{g}$) of 145 °C.

In case 1 the consolidation of two single polycarbonate sheets with 44% carbon fiber by volume was observed. Figure 10 shows the numerically and experimentally obtained temperature profiles, which are in good accordance. The dymanic temperature behavior during heating and cooling was reproduced very well by the simulation, but the target temperature of the heating or cooling processes was reached earlier than in the experiment.

For process control (and, in a further step process optimization), it is important to know when a particular temperature is reached in the core of the composite to ensure that the material is completely molten during heating and completely solidified during cooling. Here, the glass transition temperature of polycarbonate (=145 °C) was used. This temperature was reached after 10 s of heating in the experiment, while the simulation predicted a period of 11 s. During cooling, this temperature was reached 161.7 s into the experiment and after 165 s in the simulation. Based on the total process time of 250 s experiment and simulation differed by 0.4% in the heating process and by 1.32% in the cooling process.

#### 3.3.2. Cases 2, 3 and 4

Cases 2, 3 and 4 are presented together because they used the same material, layup and cooling process, and differed only in the heating temperature set at the consolidation unit (see Table 3).

As mentioned in Section 2.2.3, the temperature was recorded and analyzed at three positions within the composite for these cases, where one position was located in the core and the other two at the outer layers.

**Figure 11:**
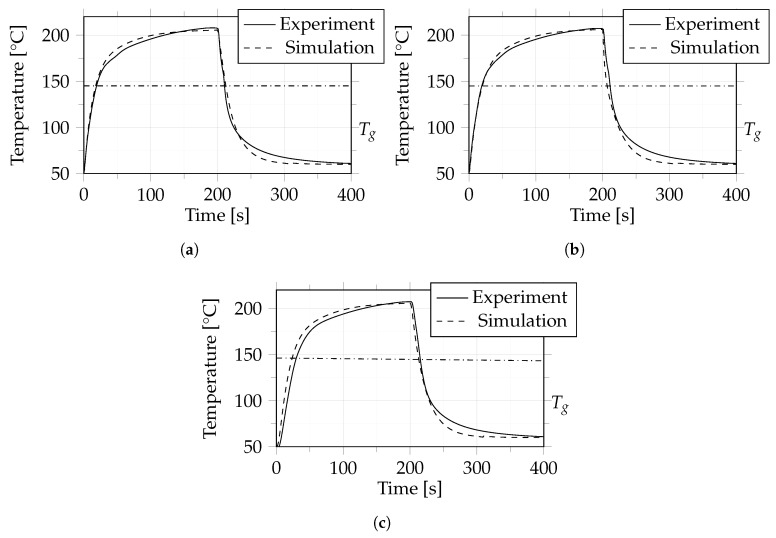
Case 2: Comparison between experiment and simulation in terms of temperature between layers 1 and 2 (**a**), 9 and 10 (**b**) and 17 and 18 (**c**). The dash-dotted line indicates the glass transition temperature ($T_{g}$) of 145 °C.

**Figure 12:**
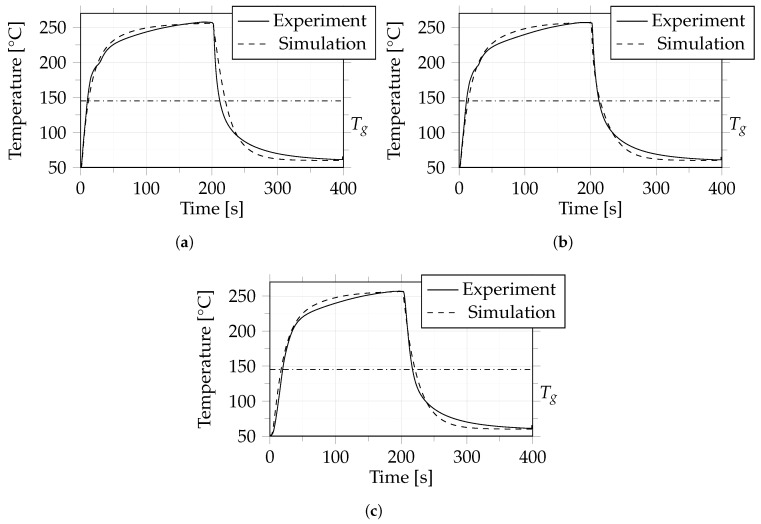
Case 3: Comparison between experiment and simulation in terms of temperature between layers 1 and 2 (**a**), 9 and 10 (**b**) and 17 and 18 (**c**). The dash-dotted line indicates the glass transition temperature ($T_{g}$) of 145 °C.

**Figure 13:**
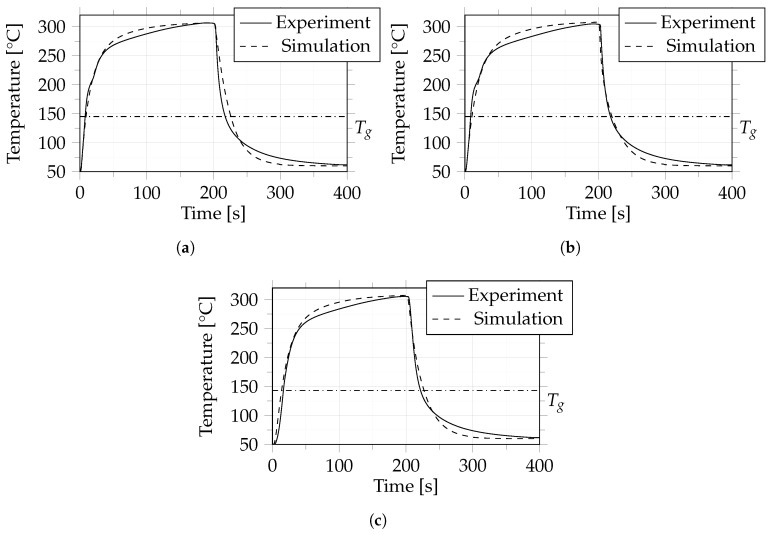
Case 4: Comparison between experiment and simulation in terms of temperature between layers 1 and 2 (**a**), 9 and 10 (**b**) and 17 and 18 (**c**). The dash-dotted line indicates the glass transition temperature ($T_{g}$) of 145 °C.

Figure 11, Figure 12 and Figure 13 show the results of a comparison between experiment and simulation. It can be seen that the difference between experiment and simulation is comparable to that in case 1, which means that the dynamic behaviour during heating and cooling was predicted very well by the simulation.

For these cases we also determined when the glass transition temperature was reached in the core (between layer 9 and 10) of the composite material during heating and cooling, the results of which are summarized in Table 4.

#### 3.3.3. Case 5

**Figure 14:**
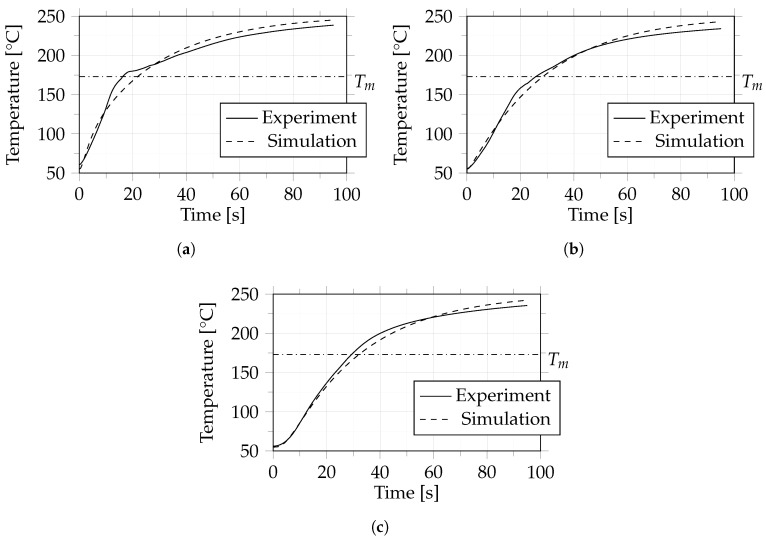
Case 5: Comparison between experiment and simulation in terms of temperature between layers 1 and 2 (**a**), 9 and 10 (**b**) and 17 and 18 (**c**). The dash-dotted line indicates the melting temperature ($T_{m}$) of 173 °C.

In order to assess the flexibility of the model in relation to the materials used, the temperature behavior of a 10-layer layup of polypropylene with 45.3% glass fiber by volume was investigated. As mentioned in Section 2.2.3 only heating from 50 °C to 250 °C was considered. The results of this experiment can be seen in Figure 14. In contrast to the cases shown above, the temperature curve in the outer layers (Figure 14a,b) shows irregularities around the melting temperature (173 °C) which are not apparent in the simulation. A possible reason for the temperature fluctuations could be the melting behavior, which was not taken into account in the simulation and was not investigated further in this work.

Comparison between experiment and simulation in terms of the time when the melting temperature was reached shows a difference of 2.84 s. Relative to the total process time of 100 s this amounts to a deviation of the simulation from the experiment by 2.84%.

As already mentioned, the experimental results show a change in the slope of the curve in the outer layers (Figure 14a,b) around the melting temperature ($T_{m}$ = 173 °C). Since the model does not consider phenomena related to crystallization, the simulation results cannot capture differences in temperature behavior between semi-crystalline and amorphous plastics.

## 4. Discussion and Conclusions

We have shown that our multi-region, multi-phase and multi-component-mixture approach is suitable for modeling the heat transfer during the consolidation process. It is robust within a range of ±20% of the thermal parameters taken from the literature and insensitive to variations in mesh resolution. Simulation results and experimental data were in very good agreement, especially in relation to the dynamic phase during heating and cooling. The simulation allows very precise estimation of when a particular temperature, such as the glass transition temperature or melting point, will be reached at the core of a composite. In relation to the total process time, deviation of the simulation from the experimental data were the following: 0.4% during heating and 1.32% during cooling for Case 1, 1.58% during heating and 0.55% during cooling for Case 2, 0.63% during heating and 0.98% during cooling for Case 3, and 0.65% during heating and 1.2% during cooling for Case 4.

Case 5 from our parameter study, using polypropylene with 45.3% glass fiber by volume, demonstrated that the model is also capable of predicting the temperature behavior of semi-crystalline matrix materials. Concerning the time when the melting temperature is reached within the core of the layup, the difference between simulation and experiment is 2.84% with respect to the total process time.

Our model, and in particular the accuracy of its predictions, enables process optimization in the form of reduction of cycle time and therefore improving the energy efficiency, and the building of digital twins. It will form the basis for implementing further models to investigate, for instance, degree of bonding, compression and squeeze-flow behavior and crystallinity. This gives the opportunity to consolidate thermoplastic composites in a more time- and energy-saving way.

## Figures and Tables

**Table 1:** Sources of the material parameters.

Parameter	Source	Value
specific heat capacity matrix $c_{p,m}$	measured	polynomial fit
specific heat capacity fiber $c_{p,f}$	litaerature	1200 J/(kg K)
specific heat capacity mixture $c_{p,M}$	measured + Equation (9)	polynomial fit
thermal conductivity matrix $\lambda_{m}$	literature	0.2 W/(m K)
thermal conductivity fiber $\lambda_{f}$	literature	0.5 W/(m K)
thermal conductivity mixture $\lambda_{M}$	Equation (9)	0.332 W/(m K)
density matrix $\rho_{m}$	measured	polynomial fit
density fiber $\rho_{f}$	literature	1790 kg/m^3^
density mixture $\rho_{M}$	Equation (9)	polynomial fit
dynamic viscosity matrix $\eta_{m}$	measured	polynomial fit
dynamic viscosity fiber $\eta_{f}$	Equation (9)	2.3 × 10^6^ Pa s
dynamic viscosity mixture $\eta_{M}$	measured	polynomial fit

**Table 2:** Material parameters for the sensitivity study.

	Original Value	−20%	+20%
specific heat capacity matrix $c_{p,m}$	1700 J/(kg K)	1360 J/(kg K)	2040 J/(kg K)
specific heat capacity fiber $c_{p,f}$	1200 J/(kg K)	960 J/(kg K)	1440 J/(kg K)
thermal conductivity matrix $\lambda_{m}$	0.2 W/(m K)	0.16 W/(m K)	0.24 W/(m K)
thermal conductivity fiber $\lambda_{f}$	0.5 W/(m K)	0.4 W/(m K)	0.6 W/(m K)

**Table 3:** Validation study process parameters: heating temperature (T_H_), cooling temperature (T_C_), heating pressure (p_H_), cooling pressure (p_C_) and cycle times for heating (t_H_) and cooling (t_C_) of polycarbonate with carbon fibers (PC-CF) and polypropylene with glass fibers (PP-GF).

	Material	Layup	T_H_	T_C_	p_H_	p_C_	t_H_	t_C_
Case 1	PC-CF	2 Sheet Layers	320 °C	80 °C	1 bar	10 bar	140 s	110 s
Case 2	PC-CF	18 Tape Layers	200 °C	60 °C	1 bar	30 bar	200 s	200 s
Case 3	PC-CF	18 Tape Layers	250 °C	60 °C	1 bar	30 bar	200 s	200 s
Case 4	PC-CF	18 Tape Layers	300 °C	60 °C	1 bar	30 bar	200 s	200 s
Case 5	PP-GF	10 Tape Layers	250 °C	–	1 bar	–	95 s	–

**Table 4:** Comparison between experiment and simulation regarding times when glass transition temperature is reached.

	Time- Experiment	Time- Simulation	Absolute Difference	Difference with Respect to Total Process Time
Case 2—heating	29.3 s	23 s	6.3 s	1.58%
Case 2—cooling	215.2 s	213 s	2.2 s	0.55%
Case 3—heating	19.5 s	17 s	2.5 s	0.63%
Case 3—cooling	217.1 s	221 s	3.9 s	0.98%
Case 4—heating	16.6 s	14 s	2.6 s	0.65%
Case 4—cooling	221.1 s	226 s	4.8 s	1.2%

## Data Availability

The data presented in this study are available on request from the corresponding author.

## Abbreviations

The following abbreviations are used in this manuscript:

AFP	Automated fiber placement
ATL	Automated tape laying
ILSS	Interlaminar shear strength
CFD	Computational fluid dynamics
